# Landscape genomics: natural selection drives the evolution of mitogenome in penguins

**DOI:** 10.1186/s12864-017-4424-9

**Published:** 2018-01-16

**Authors:** Barbara Ramos, Daniel González-Acuña, David E. Loyola, Warren E. Johnson, Patricia G. Parker, Melanie Massaro, Gisele P. M. Dantas, Marcelo D. Miranda, Juliana A. Vianna

**Affiliations:** 1 Departamento de Ecosistemas y Medio Ambiente, Facultad de Agronomía e Ingeniería Forestal, Pontificia Universidad Católica de Chile, Av. Vicuña Mackenna, 4860 Santiago, Chile; 2 Facultad de Ecología y Recursos Naturales, Universidad Andrés Bello, Republica 252, Santiago, Chile; 3 Departamento de Ciencias Pecuarias, Facultad de Ciencias Veterinarias, Universidad de Concepción, Av. Vicente Méndez 595, 3780000 Chillán, CP Chile; 4 Centro Nacional de Genómica y Bioinformática, Portugal 49, Santiago, Chile; 5 I+DEA Biotech, Av. Central 3413, Padre Hurtado, Santiago, Chile; 6 Smithsonian Conservation Biology Institute, National Zoological Park, 1500 Remount Road, Front Royal, VA 22630 USA; 7 University of Missouri St Louis and Saint Louis Zoo, One University Blvd., St. Louis, MO 63121-4400 USA; 8 School of Environmental Sciences and Institute for Land, Water and Society, Charles Sturt University, PO Box 789, Albury, NSW Australia; 9 Pontifícia Universidade Católica de Minas Gerais, Av. Dom José Gaspar 500, Coração Eucarístico, Belo Horizonte, MG Brazil; 10 Centro de Cambio Global UC, Santiago, Chile

**Keywords:** Comparative mitogenomics, Selection, Penguins, Adaptation

## Abstract

**Background:**

Mitochondria play a key role in the balance of energy and heat production, and therefore the mitochondrial genome is under natural selection by environmental temperature and food availability, since starvation can generate more efficient coupling of energy production. However, selection over mitochondrial DNA (mtDNA) genes has usually been evaluated at the population level. We sequenced by NGS 12 mitogenomes and with four published genomes, assessed genetic variation in ten penguin species distributed from the equator to Antarctica. Signatures of selection of 13 mitochondrial protein-coding genes were evaluated by comparing among species within and among genera (*Spheniscus, Pygoscelis*, *Eudyptula*, *Eudyptes* and *Aptenodytes*). The genetic data were correlated with environmental data obtained through remote sensing (sea surface temperature [SST], chlorophyll levels [Chl] and a combination of SST and Chl [COM]) through the distribution of these species.

**Results:**

We identified the complete mtDNA genomes of several penguin species, including *ND6* and 8 tRNAs on the light strand and 12 protein coding genes, 14 tRNAs and two rRNAs positioned on the heavy strand. The highest diversity was found in NADH dehydrogenase genes and the lowest in *COX* genes. The lowest evolutionary divergence among species was between Humboldt (*Spheniscus humboldti*) and Galapagos (*S. mendiculus*) penguins (0.004)*,* while the highest was observed between little penguin (*Eudyptula minor*) and Adélie penguin (*Pygoscelis adeliae*) (0.097). We identified a signature of purifying selection (Ka/Ks < 1) across the mitochondrial genome, which is consistent with the hypothesis that purifying selection is constraining mitogenome evolution to maintain Oxidative phosphorylation (OXPHOS) proteins and functionality. Pairwise species maximum-likelihood analyses of selection at codon sites suggest positive selection has occurred on *ATP8* (Fixed-Effects Likelihood, FEL) and *ND4* (Single Likelihood Ancestral Counting, SLAC) in all penguins. In contrast, *COX1* had a signature of strong negative selection. *ND4* Ka/Ks ratios were highly correlated with SST (Mantel, *p*-value: 0.0001; GLM, *p*-value: 0.00001) and thus may be related to climate adaptation throughout penguin speciation.

**Conclusions:**

These results identify mtDNA candidate genes under selection which could be involved in broad-scale adaptations of penguins to their environment. Such knowledge may be particularly useful for developing predictive models of how these species may respond to severe climatic changes in the future.

**Electronic supplementary material:**

The online version of this article (10.1186/s12864-017-4424-9) contains supplementary material, which is available to authorized users.

## Background

The mitochondrial genome (mtDNA) is under continuous selection since the 13 protein-coding genes produce polypeptide products that work in association with nuclear-encoded subunits of protein complexes involved in oxidative phosphorylation (OXPHOS) [1]. Three types of hypotheses have been proposed to explain mitogenome evolution: 1) mitochondrial OXPHOS generates both energy and heat, and environmental temperature imposes a selective trade off between ATP production and heat [2–6]; 2) risk of starvation is associated with restricted food availability, since starvation can generate more efficient coupling of energy production by OXPHOS pathway [7–10]; 3) a more recent and debated hypothesis of immune response to pathogens [11–15]. Therefore, chlorophyll (Chl), as a measurement of primary productivity [16], and sea surface temperature (SST), are important environmental variables for marine organisms that are directly related to the efficiency of the OXPHOS and relevant to understanding mtDNA gene selection patterns. As OXPHOS is the terminal stage of cellular respiration, disruption of this process will be lethal for individual cells and if it occurs widely in an individual, it will adversely impact its fitness [3]. Consequently, the mitochondrial genome on one hand evolves under continuous purifying selection (or negative selection) that removes deleterious variation from populations [17], while on the other hand, beneficial variation in extreme temperature environments may be under positive selection, increasing in frequency in a population or species. It is possible that selection may operate similarly among species in similar environments or it may drive adaptations in different genes.

Patterns of adaptation in the mtDNA of organisms have been evaluated mostly at the population level by associating each population or lineage with local climatic conditions [8, 18–21]; however, only relatively few have incorporated environmental data in the genetic analyses and generally only using nuclear genes or whole-genome data [22, 23]. Environmental temperature is the main variable that has been linked to selection in mtDNA genes [8, 18–20], along with altitude [24–26] and oxygen availability [27]. Although most studies on the role of selection on mtDNA genome evolution in vertebrate species have focused on mammals at the intraspecific level [28–31], recently there has been an increased focus on birds [32–34]. Here, we provide one of the first species-level analyses in birds linking patterns of mtDNA selection with environmental variables (temperature, chlorophyll) which are associated with the evolution of this genome. Chlorophyll (Chl) is an excellent indirect measurement of food availability because primary productivity is associated with species distributions at trophic levels, including krill and penguins [16].

Penguins inhabit a broad latitudinal distribution in the Southern Hemisphere from the tropics to the extreme cold of the Antarctic. While all penguin species can live and forage at sea in cold, nutrient-rich water (including those breeding on the Galapagos Islands), they breed on land under varying climatic conditions. The northernmost species is the Galapagos penguin (*Spheniscus mendiculus*), while the southernmost distributed species, the Adélie penguin (*P. adeliae*) and Emperor penguin (*Aptenodytes forsteri*)*,* breed in the Antarctic, south of the Antarctic Convergence (Polar Front). Emperor penguins breed on stable fast ice during the Antarctic winter when temperatures drop regularly to −50 °C. As the evolution, speciation and extinction of penguins are closely related to historical climatic changes [35–38], the broad distribution of penguins, which includes different and extreme climatic conditions, provides a unique opportunity to identify molecular genetic patterns associated with these climatic conditions.

**Fig. 1:**
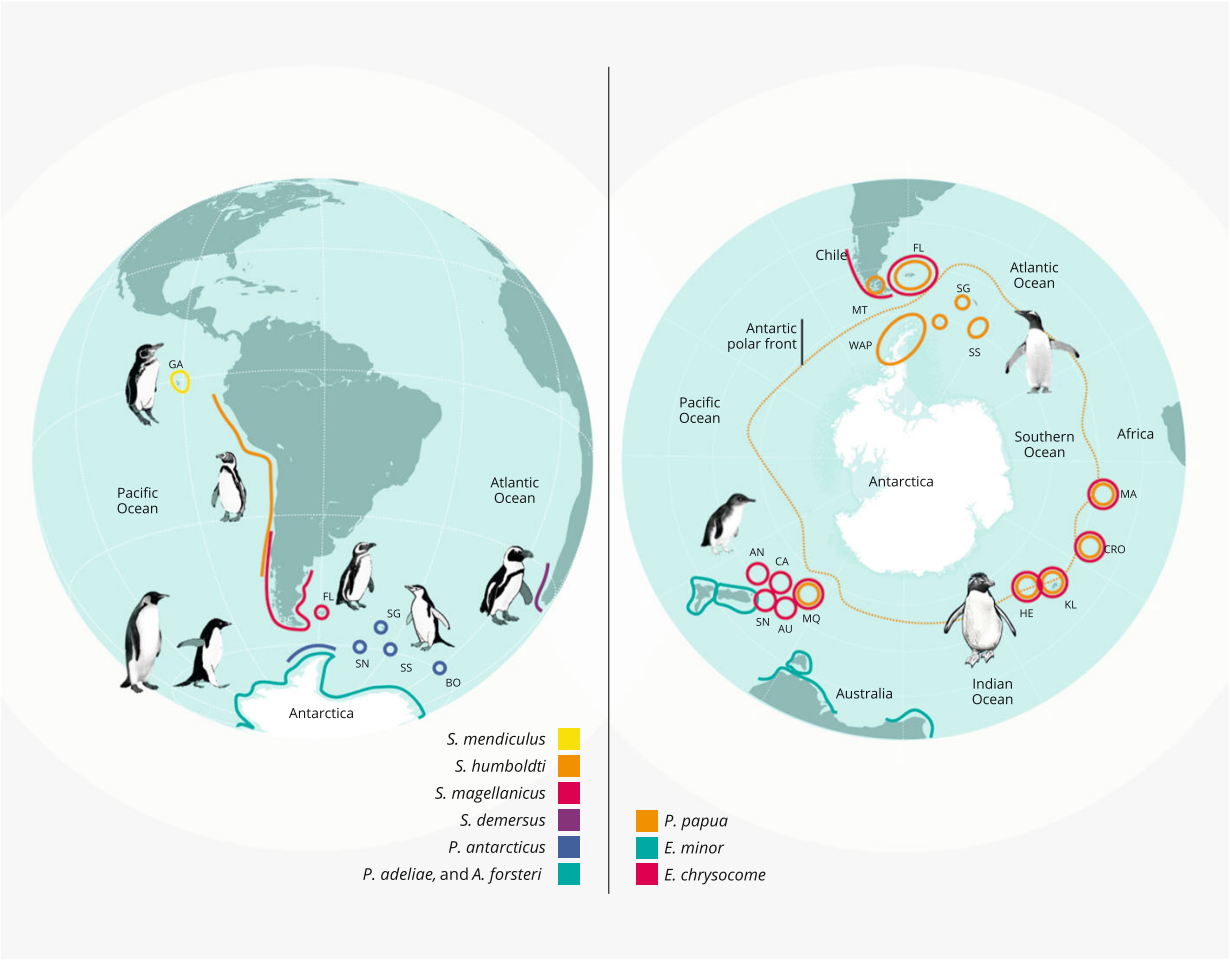
Map of studied penguin distribution. Locality where samples were collected (indicated with a star): Galapagos (red), Humboldt (blue), Magellanic (green), African (orange), Gentoo (pink), Chinstrap (dashed line), Adélie penguin (yellow). Antarctic Polar Front (yellow line), locations are indicated as: Martillo Island (MT), Falkland/Malvinas Islands (FL), South Shetland Islands and Western Antarctic Peninsula (WAP), Scotia Arc (SN, South Orkney Islands; SS, South Sandwich Islands; SG, South Georgia), Bouvet Island (BO), Crozet Islands (CRO), Kerguelen Islands (KE), Marion and Prince Edward Islands (MA), Heard Islands (HE), Macquarie Islands (MQ), Snares Islands (SN), Auckland Island (AU), Antipodes (AN), Campbell (CA). By Juan Pablo Bravo

We used Next Generation Sequencing approaches to study patterns of selection in the mitochondrial genome of penguins from two genera (*Spheniscus* and *Pygoscelis*) and correlated the results with remote sensing spatial data for each species from throughout their distribution (sea surface temperature, chlorophyll levels, and both variables combined). The four *Spheniscus* penguin species are distributed along the coast of southern Africa (African penguin *S. demersus*) and along the South America coast from Galapagos Islands in the tropics (Galapagos penguin *S. mendiculus*) to southern Patagonia (Humboldt *S. humbodti* and Magellanic penguin *S. magellanicus*) (Fig. 1). Galapagos and Humboldt penguin populations have experienced high mortality rates [39, 40] and decreased breeding success [39, 41] from increased water temperatures during El Niño Southern Oscillations (ENSO) [42]. The three *Pygoscelis* penguins breed on sub-Antarctic islands and the coast of Antarctica and they have sympatric distributions in the Western Antarctic Peninsula (WAP). The Gentoo penguin (*P. papua*) has the northernmost range in the genus, with a distribution along sub-Antarctic Islands and the Antarctic Peninsula. The Chinstrap penguin (*P. antarcticus*) has a more southern distribution, almost exclusively around the Antarctic peninsula. The Adélie penguin is the most southern and pagophilic species, with a circumpolar distribution [43]. Over the last decades climate change has impacted the WAP, increasing sea surface temperatures (SST) and changing patterns of penguin food availability (e.g. krill) [44–46]. Concurrently, Adélie and Chinstrap penguin populations have been decreasing, while Gentoo penguin populations have increased in numbers and distributions [44–46].

Historical climate changes have been linked with penguin species diversification. Severe declines in Antarctic temperature that began approximately 12 mya coincide with the divergence of the four major penguin groups, around 11–16 mya [38]. This penguin phylogeny is well characterized, making it possible to assess evidence of selection and adaptive evolution of the mtDNA and to explore possible links with environmental changes, including genes involved in penguin thermal adaptation. Sequences of entire mitochondrial genomes are available for five of the 18 extant species of penguins [47–50], including the Adélie, Chinstrap, African, Little*,* and Southern Rockhopper (*E. chrysocome*) penguins and more recently the entire genome became available for the Adélie and Emperor penguin [51]*.* The mtDNA genome of *P. antarcticus* consists of 15,972 bp and has 94% identity with its sister species *P. adeliae* [50]. Subramanian et al. [49] compared the mitochondrial genomes of modern and ancient (up to 44,000 years old) *P. adeliae*, documenting higher evolutionary rates than described earlier by phylogenetic studies. However, those studies evaluated the genome without a broader comparative approach to understand the evolution of the group and assess the evidence of signatures of selection on the mitochondrial genome.

We hypothesize that selection operates differentially on the mtDNA coding genes of penguin species in response to environmental variables. We evaluated variation in mitochondrial genomes at different taxonomic levels in seven species of penguins from South America and Africa (all four *Spheniscus* species) and Antarctica (all three *Pygoscelis* species). We then compared our results with two penguin species from temperate and sub-Antarctic regions (*E. minor and E. chrysocome*) and with *A. forsteri*, which survives and breeds during the most-extreme cold conditions of the Antarctic winter. We evaluated evolutionary rates and signatures of natural selection for all 13 mtDNA protein-coding genes and across the mtDNA genome phylogeny for the penguin species. Lastly, we assessed the genetic data for each gene against the spatial environmental data (sea surface temperature, chlorophyll and both variables combined) from remote sensing and GIS databases for each species along its distributional range.

## Methods

We sequenced the entire mtDNA genome of 12 individuals from 6 species of penguins: the Galapagos (*S. mendiculus*, *n* = 1), Humboldt (*S. humboldti*, *n* = 2), Magellanic (*S. magellanicus*, *n* = 3), Adélie (*P. adeliae*, *n* = 2), Gentoo (*P. papua*, *n* = 2) and Chinstrap penguins (*P. antarcticus*, *n* = 2). We sequenced individuals from different latitudes or the extreme limits of their distribution (Fig. 1, Tables 1, 2). Adélie samples were obtained from the two divergent lineages identified by Ritchie et al. [52] using D-loop mtDNA.

**Table 1:** Latitudinal range, adult body mass, adult length and broad climatic preference for each penguin species [79, 110], [Vianna pers. comm.]

Genus	Species	Common Name	Geographic range		Body Mass (kg)		Length (cm)	Climate
			Latitude (S)		range	Average		
*Pygoscelis*	*antarcticus*	Chinstrap Penguin	54°	64°	3.5–5.5	4.1	61–76	Temperate/Polar
	*papua*	Gentoo Penguin	46°	65°	3.3–8.8	6.1	56–83	Temperate/Polar
	*adeliae*	Adélie Penguin	54°	75°	4.6–5.4	5	46–71	Polar
*Spheniscus*	*mendiculus*	Galápagos Penguin	0°	0°	1.4–2.6	2	46–55	Tropical
	*humboldti*	Humboldt Penguin	5°	42°	4.2–5.0	4.6	67–72	Tropical/Temperate
	*magellanicus*	Magellanic Penguin	40°	55°	3.2–5.2	4	48	Temperate
	*demersus*	African Penguin	25°	33°	2.7–3.4	3.1	60–70	Subtropical/Temperate
*Eudyptula*	*minor*	Little Penguin	30°	47°	0.7–1.3	1.2	33	Temperate
*Eudyptes*	*chrysocome*	Southern Rockhopper Penguin	46°	54°	2–3.8	3.4	45–55	Temperate
*Aptenodytes*	*forsteri*	Emperor Penguin	64°	77°	22-40	28	120	Polar

**Table 2:** Details of the individuals sequenced

Genus	Species	Locality	Geographic position		Total genomic reads	MtDNA		Coverage	Genome lenght (bp)		Genbank
			Latitude (S)	Longitude (W)		Total Reads	Used Reads		all genes	+ D-loop	
*Pygoescelis*	*P. antarcticus*	Antarctica, South Shetland Islands, Narebski base	62° 13’	58° 45’	80,496,366	15,788	11,347	36	15,491	16,664	KU356673
		Antarctic Peninsula, Kopaitic	63° 19’	57° 54’	52,990,428	9298	6359	20	15,490	16,653	KU356674
	*P. papua*	Elephant Island, South Shetlands Islands	61° 06’	55° 09’	7,341,640	2630	1647	7^a^	–	–	–
		Antarctic Peninsula, Gabriel Gonzalez Videla base	64° 48’	62° 51’	158,565,602	34,092	25,947	82	15,500	16,704	KU356677
	*P. adeliae*	Antarctica, South Shetland Islands, Arctowski base	62° 09’	58° 28’	52,997,551	8162	5049	16	15,493	16,764	KU356675
		Antarctica, Ross Sea, Cape Crozier	77° 29’	69° 20’	68,998,340	13,000	7956	26	15,493	16,765	KU356676
*Spheniscus*	*S. mendiculus*	Galapagos Islands, Isabela Island	00° 35’	91° 05’	69,908,826	13,006	7360	24	15,516	16,367	KU361807
	*S. humboldti*	South America, Peru, Punta San Juan	15° 21’	75° 11’	64,663,956	21,786	13,908	43	15,519	16,658	KM891593
		South America, Chile, Cachagua	32° 35’	71° 27’	55,087,204	14,606	9269	28	15,519	16,660	KU361805
	*S. magellanicus*	South America, Chile, Chiloé Island, Puñihuil	41° 55’	74° 02’	61,046,791	11,456	5984	19	15,520	16,427	KU361804
		South America, Chile, Magdalena Island	52° 51’	70° 36’	54,372,256	11,108	6490	20	15,514	16,659	KU361806
		South America, Argentina, Puerto Deseado	47° 45’	65° 43’	67,969,432	10,640	5483	18	15,520	16,549	KU361803

^a^Genome not included in all analyses due the low coverage

### SOLiD sequencing

Genomic DNA was isolated from blood samples obtained from all penguins and preserved in ethanol using the salt method [53]. DNA from six individuals of the three species was quantified and quality checked with Invitrogen’s PicoGreen®assay kit (fluorometry). Genomic sequencing in ABISOLiD™ 5500 XL was performed at Omics Solution, a Next Generation Sequencing facility. DNA was desalted then concentrated using standard EtOH/sodium acetate precipitation at 20 °C for 2 h, followed by two 70% EtOH washes. Then the pooled DNA was re-dissolved in low TE as per standard protocol for ABI SOLiD sequencing of genomic DNA fragment libraries. DNA samples were sheared in Covaris™S220 System, which fragments the input DNA, by sonication, into small fragments with a mean size of 160 bp.

The fragmented DNA was then purified with SOLiD Library Column Purification Kit, and libraries were prepared according to standard SOLiD protocols. Fragment libraries for the twelve penguins were prepared separately, P1 and P2 adapters were ligated, and each sample was tagged with a different barcode (a known sequence of ten bp in adapters). Prepared libraries were quantified by real-time PCR on a Light Cycler®Nano (Roche) using Invitrogen’s Quantification Kit for SOLiD. The double stranded library was added at a concentration of 0.2 pg/μL to the emulsion with 2400 million beads, according to manufacturers’ instructions. Thirty percent of the beads were P2 positive (contained amplified library fragments) before enrichment and 90% of the beads were P2 positive after enrichment, yielding 790 million beads deposited on the Flow Chip. Library beads were sequenced on a SOLiD™ 5500 XL using standard chemistry for paired-end fragment libraries and 75–35-bp read lengths.

### SOLiD sequence alignment

The color-space reads (di-base encoded) were aligned with LifeScope™ (Applied Biosystems) using the genome assembly of *P. adeliae* [51] as a reference. The reference was translated into color-space with the aim of mapping the reads. The color-space reads helped to improve the quality of each base call, since each base was read twice during the sequencing step.

The consensus sequence was built from the binary alignment map (BAM) files obtained in the previous step. We used SAMtools [54] to obtain all bases mapped to each position, BCF tools to get the most probable genotype per position, and VCF utils to build the consensus sequence in FASTQ format. The FASTQ file was then converted to FASTA using SEQTK.

### Mitochondrial sequence assembly

The mitochondrial genomes were assembled with the Denovo2 pipeline developed by Life Technologies using Velvet assembler version 1.2.09 [55] in color-space mode. ASiD (Assembly Assistant for SOLiD) was used to fill gaps between contigs that formed scaffolds and to perform a color-space to base-space translation for the final assembly. Individual genes were selected from each of the penguins’ contigs using Sequencher software 5.1 (Gene Codes Corp., MI) and annotated by homology with genes of mitogenomes of the penguins previously published [48, 51, 56, 57]. Due to the low number of reads from one individual of *P. papua* (Table 2), this individual was not included in the data analyses. All 11 mitogenome were submitted to GenBank (accession numbers KM891593; KU361803-KU361806; KU356673-KU356677).

### Molecular evolution and selection

Sequences were aligned and polymorphic sites were confirmed by visual review of the chromatograms using Sequencher v. 5.1. (Gene Codes, Ann Arbor, MI, USA) and multiple alignments were done using ClustalX v. 2.1 [58]. For interspecific data analysis we compared the eleven mtDNA genomes with four other penguins genomes: *S. demersus* obtained from KwaZulu-Natal Province of South Africa (African penguin) [56], *E. minor* obtained from Nelson in New Zealand (Little blue penguin) [48], *E. chrysocome* (Rockhopper penguin) [57] and *A. forsteri* (Emperor penguin) [51].

The number of indels, polymorphic sites (S), nucleotide diversity (π), synonymous mutation rates (Ks), nonsynonymous mutation rates (Ka) and the Ka/Ks (or ω) ratio using the DNAsp v. 5 [59] were estimated for all 13 protein-coding genes from the complete mtDNA. For analyses of the reading frame of the genes, such as Ka/Ks, only the *ND3* gene was considered a different reading frame as described by Mindell et al. [60]. All Ka and Ks estimates and corresponding ratios were obtained between pairwise species within *Spheniscus* and *Pygoscelis* genera, as well as between all five genera (Additional file 1 Table S1). Since we sequenced several species of *Spheniscus* and *Pygoscelis* genera, the values between genera were averages of the pairwise species Ka and Ks values for the genera comparison. We also calculated the pairwise distance between all 15 penguins studied here (10 species) for all 13 concatenated genes of the mtDNA genomes using the Maximun Likelihood model (Additional file 2 Table S2) with Mega v. 6.06 software [61, 62] and Arlequin software in R package [63, 64]. All positions containing gaps were eliminated, remaining a total of 10,874 positions in the final dataset.

Phylogenetic reconstruction was used to evaluate selection on each codon, gene and phylogenetic branch. We combined each of the 13 protein-coding genes from the mtDNA genome from the studied penguin species into a concatenated data set of 11,409 bp. The phylogeny was also estimated excluding the *ND6* gene (a total of 10,886 bp), which is encoded by the light-strand [49] and has a significantly different base composition from the heavy-chain [65]. Phylogenetic relationships were inferred using Bayesian inference methods (BA) with Markov chain Monte Carlo (MCMC) sampling as implemented in MrBayes v. 3.1.2 [66, 67]. The evolutionary model selected GTR + I + G, using jModelTest v. 0.1.1 [68] and Akaike Information Criterion (AIC). The Mr. Bayes analyses were run for one million generations while sampling every 100 generations. At this point the standard deviation of split frequencies was <0.01, indicating that both runs had converged. Phylogenetic reconstructions were visualized using Figtree v. 1.2.2 [69]. Although positive selection is frequently inferred by genetic data analysis as Ka/Ks > 1 (and purifying selection by Ka/Ks < 1) [70], a gene with Ka/Ks lower than 1 may show signature of positive selection at codon level. Evidence for signatures of selection at codon sites was evaluated for each gene with the concatenated data set using the BA phylogeny and the selected model of evolution without outgroups with the three algorithms available in HyPhy software [71–73]. For comparison, we used the Single Likelihood Ancestral Counting (SLAC), Fixed-Effects Likelihood (FEL) and the Random Effects Likelihood (REL) methods using the best fit nucleotide model [71]. The single likelihood ancestor counting (SLAC) method is based on weighting across all possible ancestral reconstructions, or employs sampling from ancestral reconstructions. The fixed effects likelihood (FEL) method estimates nonsynonymous and synonymous substitution rates at each site. Random effects likelihood (REL) models variation in nonsynonymous and synonymous rates across sites based on a predefined distribution, where a Bayesian approach are used to infer selection pressure at an individual site [71]. We also performed the branch Site REL [72] analysis in HyPhy software to estimate branch-specific episodes of positive selection along the phylogeny.

### Environmental data and species distribution

Seasonal Chlorophyll concentrations (Chl) and Sea Surface Temperatures (SST) were acquired from Sea-viewing Wide Field-of-view Sensor (SeaWiFS) and Moderate Resolution Imaging Spectroradiometer (MODIS-Aqua) global seasonal databases that are based on a spatial resolution of 9 × 9 km [74]. The standard SeaWiFS chlorophyll product, ChlO_C4_, is estimated based on an empirical maximal band-ratio algorithm that uses remote sensing reflectance measurements at 443, 490, 510 and 555 nm [75]. This algorithm relates the maximum of three remote sensing reflectance band-ratios (443/555, 490/555 and 510/555) to field measures of sea chlorophyll concentration [76] SST is based on MODIS-calibrated mid- and far-infrared radiances (IR), estimated using an algorithm that exploits differences in atmospheric transmissivity in the different IR bands [77]. The reliability of SST data is supported by its high correlation with sea surface temperatures measured during both oceanographic cruises and at local stations, with a slight positive bias at higher temperatures [78]. Here we use Chl and SST as proxies for the environmental conditions along the distributional range of each penguin species.

**Fig. 2:**
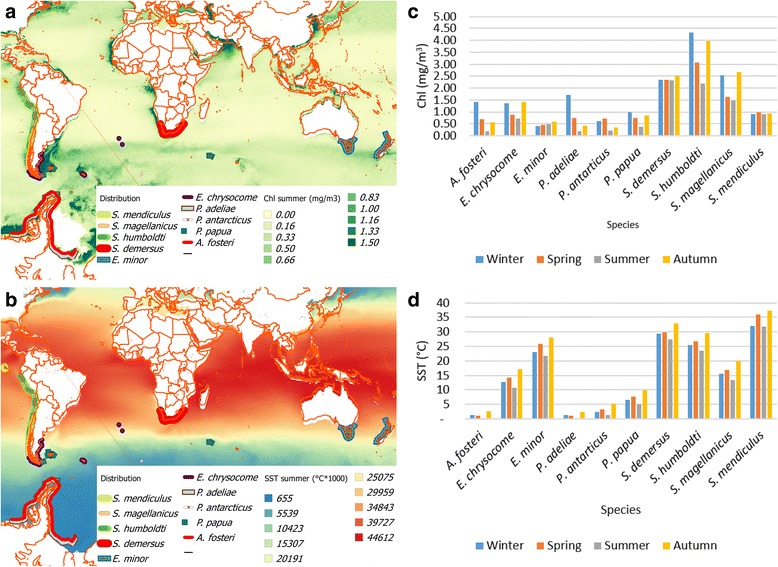
Chlorophyll (Chl) and Sea Surface Temperature (SST) along most of the 10 species breeding distribution. Data were obtained from SeaWifs [75] and MODIS sensors [74]. Map of (**a**) Chl and (**b**) SST during the summer; and graph of the four seasons (**c**) Chl and (**d**) SST for each penguin species

Satellite data (Chl and SST) were processed and saved in image format using SAGA - System for Automated Geoscientific Analyses (SAGA Development Team 2008). Penguin at-sea distribution during breeding were obtained from Borboroglu and Boersma [79] and digitized in vector format using a Geographic Information System QGis 2.6 [80]. Mean and standard deviation of seasonal Chl and SST products were obtained for each species by intersecting seasonal images with the vector files representing the geographical distribution of each species (Fig. 2). All data were re-scaled into a range from 0 to 1 to be comparable the Chl (Fig. 2a, c) and SST data (Fig. 2b, d).

Using this information, between-species pairwise Euclidian distances for Chl, SST and Chl-SST (combinated environmental distance COM) were calculated using the equation Eq. (1):

1 $$ {D}_{xy}=\sqrt{{\left({W}_x-{W}_{y\Big)}\right)}^2+{\left({A}_x-{A}_y\right)}^2+{\left({SP}_x-{SP}_x\right)}^2+{\left({S}_x-{S}_y\right)}^2} $$

where *D*_*xy*_ is the Chl, SST or COM distance between two penguin species *x* and *y* based on the seasonal variability of Chl and SST. *W*, *A*, *SP* and *S* are the values of Chl, SST or COM in the winter, autumn, spring and summer season respectively.

### Mantel test and GLM models

Mantel tests and GLM models (Generalized Linear Model) were implemented to explore the relationships between distance matrices of genetic parameters (*π* and *ka/ks*) and environmental parameters (Chl, SST and COM) as explanatory variables [81, 82]. To study the relationship between genetic patterns and the environmental parameters (the distances across Chl, SST and COM dimension), the Mantel matrix distances were divided into three sub-matrices (Example in Additional file 3 Table S3), each describing the patterns between pairs of species within a bounded interval of Chl, SST and COM distances [81]. These bounded intervals consisted of a) short pairwise distances corresponding with the minimum to 33% of the data distance, b) middle distances corresponding with pairwise distances between 33% and 66% of the data distance, c) large distances consisting of the largest 33% of the pairwise distances and d) all pairwise data. For Mantel tests, *p-values* were obtained based on 10,000 simulations. Normality of the data (Ka/Ks and π) was tested applying the Shapiro-Wilk test. The GLMs *p-values* and AICs were calculated considering a Gaussian error structure for all data under normal distribution and gamma error structure for data without normal distribution. All statistical analyses were implemented using R packages [64].

## Results

### MtDNA genomes of penguins

The number of reads for each of the 12 genomes ranged from 52,990,428 to 158,565,602. One individual of *P. papua* was excluded because of insufficient reads (7,341,640). The number of reads attributed to mtDNA genomes ranged from 5049 to 25,947 (1647 for *P. papua*) and the percent coverage varied from 16 to 82 (Table 2). We recovered the full sequence from all mtDNA regions except the repetitive region of the D-loop.

These are the first reported near-complete mtDNA genomes of *S. humboldti*, *S. magellanicus*, *S. mendiculus* and *P. papua*. The complete mitochondrial genome, without the *D*-loop, varied between 15,514 bp and 15,520 bp among the three *Spheniscus* ssp. and between 15,490 bp and 15,500 bp among the three *Pygoscelis* species. Including the partial D-loop, lengths varied between 16,367 bp and 16,660 bp among the three *Spheniscus* species, and between 16,653 bp and 16,765 bp among the three *Pygoscelis* species. These mt-genome lengths are comparable to the mtDNA genomes of *P. antarcticus* (15,972 bp), *S. demersus* (17,346 bp), *E. minor* (17,611 bp), *E. chrysocome* (16,930 bp), and *P. adeliae* (17,486 bp).

**Fig. 3:**
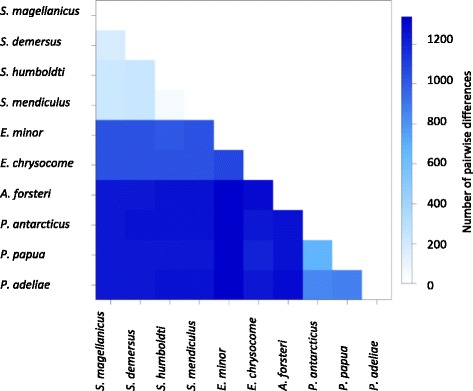
Pairwise differences between the penguin mitogenomes

The lowest evolutionary divergence among species was between *S. humboldti* and *S. mendiculus* (0.004), with six of the thirteen genes being 100% identical (*COX1*, *COX*2, *ATP8*, *ATP6*, *ND3*, *ND4L*), followed by *S. demersus* and *S. magellanicus* (0.010). The highest divergence was observed between *E. minor* and *P. adeliae* (0.097, Fig. 3, Additional file 2 Table S2).

The *ND6* and 8 tRNAs are transcribed from the light strand, while the other 12 protein coding genes and 14 tRNAs and two rRNAs are positioned on the heavy strand. This pattern of transcription has been described for *P. adeliae*, *S. demersus* and other bird species [49, 56, 83]. Indels, when comparing all ten species, were observed in five of the thirteen mtDNA genes (*ATP8, CYTB, ND4, ND5* and *ND6*). All indels comprised three consecutive bases, such as for *Pygoscelis* and *A. forsteri* at *ATP8*; for *E. chrysocome* at *CYTB*; for *A. forsteri* at *ND4* and *ND6*; and for *E. minor* at *ND5*.

At the interspecific level, higher diversity was found in NADH dehydrogenase genes (Table 3), among which *ND6* was the most diverse between all species (π = 0.11385), followed by *ATP8* (π = 0.11203). The cytochrome c oxidase genes (*COX2, COX3* and *COX1*) were the least diverse among the mtDNA genes (π = 0.05816, 0.06 and 0.07417, respectively). Comparing the two genera, *ND6* was also the most diverse among *Pygoscelis* (π = 0.0763), while *ND1* was the most diverse among the *Spheniscus* (π = 0.02152) species (Table 3). *ND3* was the second most diverse among *Pygoscelis* (π = 0.0733), however, it had the lowest nucleotide diversity among *Spheniscus* (π = 0.0622). The numbers of polymorphic sites were higher within *Pygoscelis* (S = 1197) compared to *Spheniscus* (S = 364).

**Table 3:** Interspecific diversity across whole data set (all 15 penguins of 10 species) for all 13 protein-coding genes

MtDNA			Genera				Among all species	
Gene Complex Length (bp)			*Pygoscelis*		*Spheniscus*		Data set	
			S	π	S	π	S	π
ND1	I	978	108	0.06074	49	0.02152	262	0.08941
ND2	I	1039	115	0.06141	36	0.01457	305	0.09337
COX1	IV	1551	161	0.05642	32	0.01038	344	0.07417
COX2	IV	684	61	0.04766	13	0.00919	144	0.05816
ATP8	V	165/168	17	0.05833	4	0.01212	52	0.11203
ATP6	V	684	68	0.05468	23	0.01682	176	0.08619
COX3	IV	784	56	0.03852	21	0.01215	157	0.06
ND3	I	352	46	0.0733	6	0.00622	89	0.08423
ND4L	I	297	27	0.04714	6	0.01058	71	0.08325
ND4	I	1379/1382	163	0.06425	51	0.01796	369	0.08703
ND5	I	1818/1821	180	0.05369	55	0.01435	502	0.08794
CytB	III	1143/1146	123	0.05836	32	0.01267	285	0.08026
ND6	I	519/522	72	0.0763	36	0.0211	177	0.11385
Overall		11,408	1197		364		2933	

### Gene evolution and natural selection

**Fig. 4:**
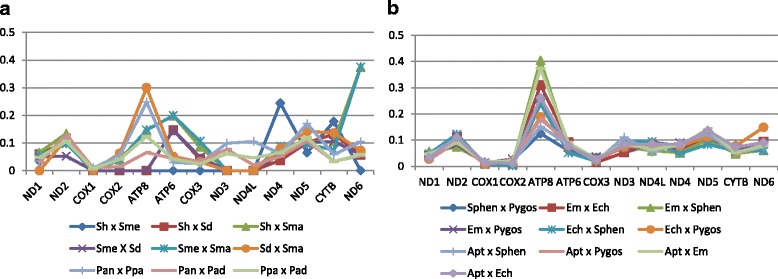
Pairwise Ka/Ks comparison of sequence divergence for the 13 mitochondria protein-coding genes. **a**- Ka/Ks ratio between species within the *Spheniscus* and *Pygoscelis* Genera; **b**- Ka/Ks ration between all species of different genera. *Spheniscus* (Sphen): *S. humboldt* (Shu), *S. magellanicus* (Sma), *S. mendiculus* (Sme), *S. demersus* (Sde). *Pygoscelis* ssp.(Pygos): *P. antarcticus* (Pan), *P. papua* (Ppa), *P. adelie* (Pad). *Eudyptula minor* (Emi), *Eudyptes chrysocome* (Ech) and *Aptenodytes forsteri* (Apt)

The general pattern of Ka/Ks ratios for the different genes was similar between pairwise species at inter-genera comparisons (*Spheniscus, Pygoscelis, Eudyptula, Eudyptes, Aptenodytes;* Fig. 4; see Additional file 1 Table S1). The Ka/Ks ratios were all <1 (values under 1 suggest negative selection and over 1 positive selection using this method). *ATP8* had the highest values and variability of Ka/Ks ratio among all species comparison between genera (Fig. 4) with values between 0.1 and 0.4. *ATP8* Ka/Ks ratios were lower between *Pygoscelis* and *Spheniscus* (0.09 to 0.15), and between *A. forsteri* and *Spheniscus* (0.14 to 0.16). High *ATP8* Ka/Ks ratios were observed between *E. minor* and most other genera (0.24 to 0.41).

Markedly low values of Ka/Ks ratios were found for within and between pairwise genera comparisons and were observed for *COX1*, followed by *COX2, COX3* and *ND1*, suggesting negative selection (Fig. 4). In the intragenus comparisons, the Ka/Ks ratios were zero for *COX1*, *ND3* and *ND4L* among all *Spheniscus* species and were also low for *COX2*, *ND1* and *COX3*. Similarly, within *Pygoscelis* comparisons, Ka/Ks ratios were lowest for *COX1*, followed by *COX3, COX2, ND1* and *ATP6*. Therefore, *COX1* had a signature of purifying selection for amino acid sequence conservation for all pairwise comparisons among penguin genera and species.

**Fig. 5:**
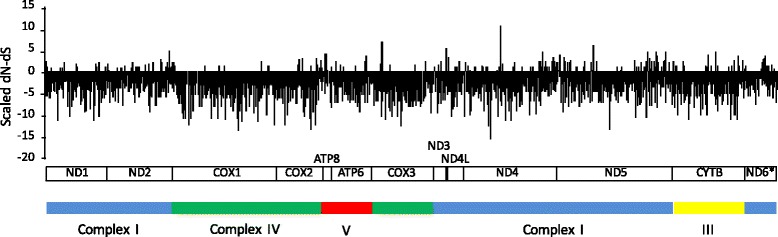
Signatures of selection for each codon for 13 mtDNA genes using SLAC method. Positive (positive values) and negative selection (negative values) are shown. ND6* is transcribed from the light strand

**Fig. 6:**
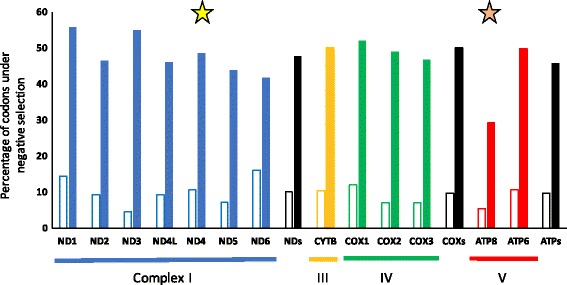
Significant negative selection for the codons of 13 mtDNA genes using SLAC and FEL methods. Dark cells: SLAC analysis. Empty cells: FEL analysis. *ND6* is transcribed from the light strand. Each star represents codons under significant positive selection as determined by SLAC (yellow star) and FEL (orange star) in ND4 and ATP8

Similar results were detected using the SLAC, REL and FEL algorithms. Overall, significant levels of positive selection were detected for 69 different codon sites in four genes (*COX1*, *ATP8*, *ND4L, ND4*). Only one codon was under positive selection for *ND4* using SLAC (codon 187) and one codon for *ATP8* (codon 36) using FEL. We detected a large number of codons under positive selection for *COX1* (a total of 39 codons) and 28 codons for *ND4L* using REL. Although REL methods have higher power and are more computationally intensive than SLAC methods of detecting selection, they have higher rates of false positives with small data sets [71, 84]. FEL approaches appear to be more robust with fewer sequences than REL and SLAC that may underestimate the substitution rate [71]. Indeed, no codon had positive and significant values by SLAC for *ATP8*. The largest number of codons with positive, but not significant signatures were identified for this gene using this method (18% of codons), in contrast with patterns observed in other genes (1.4% in *COX1* to 11% in *ND2, ND5* and *ND6*, Figs. 5, 6). Low values of negative selection were obtained for *ATP8* and *ND4L* using SLAC and FEL, and the most significant values were obtained for *COX1* (Fig. 6). We identified 375 codons with significant negative selection using SLAC algorithms and 1787 codons using FEL algorithms. All of the codons identified by SLAC overlapped with those identified using FEL algorithms. We did not detect lineages under positive selection using a Branch REL analysis based on the phylogeny.

### Environmental data

Chlorophyll concentration (Fig. 2c) was high in the area occupied by *S. humboldti*, and varied highly among seasons (2.2 mg/m^3^ and 4.3 mg/m^3^ in winter and summer respectively). Chlorophyll concentrations in the distributional range of *S. demersus* were also high, but more stable (from 2.3 mg/m^3^ to 2.5 mg/m^3^). Highest SSTs were recorded throughout the range of *S. mendiculus* (32 °C and 37 °C during winter and summer respectively), followed by lower SSTs throughout areas occupied by *S. demersus* and *S. humboldti* (27.4–32.8 °C; and 23.6–29.6 °C during winter and summer respectively), with the lowest SST in areas where *P. adelie, A. fosteri* and *P. antarcticus* are distributed (0.5 °C and 5.2 °C during winter and summer respectively) (Fig. 2d).

Nucleotide diversity (π) was not significantly correlated with environmental data at different spatial distances in any of 13 mtDNA genes for Mantel test (Additional file 4 Table S4). The Ka/Ks ratio was related with environmental data at different spatial scales for four genes supported by the significant values of Mantel test and GLM (*ND4, ND3, ND5* and *Cytb*; Table 4) and three other genes only for GLM (ND1, COX1 and ATP8). A significant positive correlation between an environmental variable and Ka/Ks ratio for a specific mtDNA gene may suggest that species inhabiting similar environments undergo adaptation processes for the same gene (similar protein reflected by Ka/Ks) and when inhabiting different environments undergo different gene adaptation processes (different protein reflected by Ka/Ks). The highest significant correlation was between SST and Ka/Ks ratio for *ND4* (Mantel, *p*-value: 0.0001; GLM, *p*-value: 0.00001) for all distances. Although, chlorophyll concentration was significantly correlated with Ka/Ks ratio of *ND1, COX1, ND5* for GLM, only *COX1* showed signature of selection and low *p*-value for Mantel (*p*-value: 0.063) at short distances. The combined environmental data (COM) were correlated with the Ka/Ks ratio for three genes supported by significant Mantel test and GLM: *ND3* (Mantel, *p*-value: 0.0304; GLM *p*-value: 0.0188), *ND5* (Mantel, *p*-value: 0.0232; GLM *p*-value: 0.0095), and *Cytb* at short distance (Mantel, *p*-value: 0.0463; GLM *p*-value: 0.0103) and middle distance (Mantel, *p*-value: 0.0064; GLM *p*-value: 0.0112). All significant *p*-values for the Mantel test were also significant for GLM (Additional file 4 Table S4). Although we detected few significant correlations between Ka/Ks ratio and environmental variables, we cannot reject the possibility that these results are influenced by other variables not evaluated here.

**Table 4:** Significant values of GLM and Mantel Test for Ka/Ks ratio for each gene and environmental data

Genes	Environmental variable	Class distance	Ka/Ks					
			Mantel				GLM	
			*Expectation*	*Observation*	*P*	*Variance*	*AIC*	*P*
*COX1*	CHL	0–33	0.0000	0.2216	0.0631	0.0162	−122.01	0.0298*
*ND3*	COM	33–66	−0.0014	0.2642	0.0304*	0.0125	−53.22	0.0188*
***ND4***	**SST**	**0–100**	**−0.0006**	**0.2978**	0.0001*	**0.0029**	**−248.99**	0.00001*
*ND5*	COM^1^	0–100	0.0000	0.1312	0.0232*	0.0038	−222.34	0.0095**
*CYTB*	COM	0–33	−0.0008	0.2476	0.0463*	0.0149	−63.94	0.0103*
	COM^1^	33–66	0.0013	0.4184	0.0064*	0.0137	−72.91	0.0112*

*significant values; bold the gene with lowest *p*-value; ^1^negative correlation

Nucleotide diversity among penguin species was not correlated with the environmental variables (no significant value for Mantel test), nevertheless Ka/Ks ratios were significantly correlated. This supports the premise that broadly, genetic diversity of mtDNA genes are conserved, but that specific signals of selection related to environmental variables could be identified by comparative estimation of Ka/Ks ratios among genes.

**Fig. 7:**
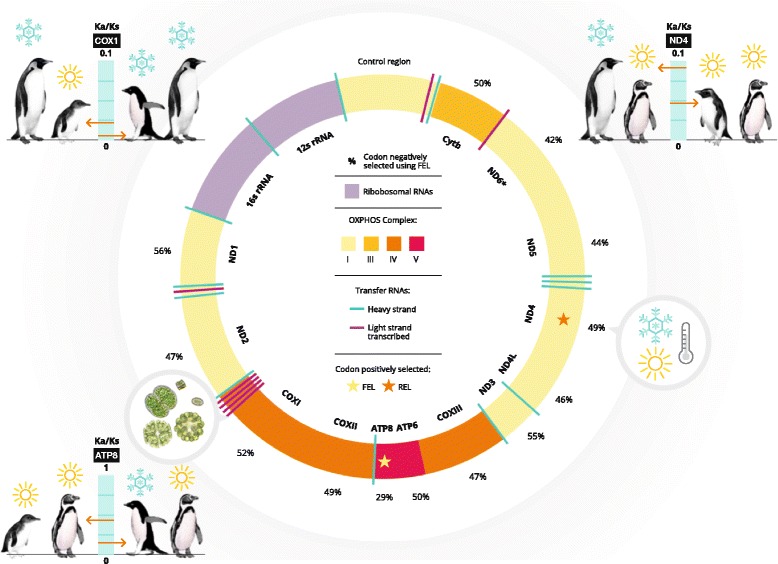
Results of signature of selection in the penguin mtDNA genome. Ribosomal RNAs (blue), Transfer RNAs (green and purple lines), Protein-coding genes with the oxidative phosphorylation system complex are represented by different colors. ND6* is transcribed from the light strand. Percentage of codons under negative selection is shown for each gene and numbers in blue highlight the relatively low percentage for ATP8. Each star represents codons under significant positive selection as determined by FEL (yellow star) and REL (orange star) in ATP8 and ND4 genes. Ka/Ks ratio for ND4 is correlated with superficial surface temperature and COX1 for chlorophyll levels. The four graphics around the genome indicate with red arrows the highest and lowest for Ka/Ks ratio comparison between species from different genus for each of the four genes under selection. COX1 and ND4 highest Ka/Ks values are associated with pairwise ratio between species from different climates, and lowest among those from similar climates. With ATP8, highest Ka/Ks values were associated with pairwise ratios between species from similar climates and lowest among those from from distinct climates. Scale for graphic of ND3 and ATP8 genes are Ka/Ks from 0 to 1, and for COX1 and ND4 are from 0 to 0.1. By Juan Pablo Bravo

Assessing genes under selection, *ND4* and *COX1* had the highest Ka/Ks ratio between species from distinct environments and the lowest ratios between species from the same environment (Figs. 4, 7). In contrast, *ATP8* had the highest Ka/Ks ratio between similar environments and the lowest between different ones.

## Discussion

Our analyses revealed strong and contrasting signals and selection patterns in the coding mtDNA genes of closely related penguin species, some of which correlated with patterns of sea surface water temperatures and chlorophyll concentrations. We identified a widespread signature of purifying selection (Ka/Ks < 1) across the mitochondrial genome, consistent with expected patterns if purifying selection were constraining the mitogenome evolution to maintain the OXPHOS proteins and functionality [20, 70]. These patterns included a signature of purifying selection in *COX* genes, especially *COX1*, which had Ka/Ks ratios close to zero for all comparison among species and genera. In contrast, one codon under positive selection was evidenced for *ND4* and one for *ATP8* (SLAC and FEL analysis respectively). Sea surface temperature (SST) was strongly correlated with the Ka/Ks ratio of *ND4* and chlorophyll concentration was correlated with Ka/Ks ratios of *COX1*. The Ka/Ks ratio patterns for *ATP8* varied widely among genera and there was only weak or no evidence of a correlation between genetic diversity of *ATP8* and measured environmental factors. These results identify mtDNA candidate genes under selection which could be involved in broad-scale adaptations of penguins to their environment.

### Evidence linking selection and gene function

#### NADH dehydrogenase

In metazoans, mitogenome complexes I through V are physically positioned relative to each other in accordance with their function, with the complex II genes residing in the nucleus [85]. A positively selected codon was detected by SLAC model on the mitogenome of penguins in the Complex I or *NADH* dehydrogenase gene (*ND4*). Complex I through IV generate the proton gradient in the intermembrane space of mitochondria. Complex I is the main entrance point of electrons and the largest complex of the respiratory chain. It catalyzes the transfer of two electrons from NADH to ubiquinone and translocates four protons across the inner mitochondrial membrane [7]. Complex I consists of different protein subunits, with fourteen homologous subunits present in all organisms, and which constitute the enzyme.

ND1 and ND2 are situated at the connection between the peripheral and the membrane arm, whereas ND4 and ND5 are at the distal end [86, 87]. If ND2, ND4 and ND5 are the actual proton pumping devices [86], mutations in these genes may alter the efficiency of the proton-pumping process. The concentration of codons under positive selection in specific domains of the mitogenome are very likely to be related to protein function [20, 88] and a meta-analysis of 237 vertebrates showed that most of the genes under positive selection were located in Complex I [89]. *NADH* dehydrogenase genes have also shown signatures of positive selection at a population level in birds (*Parus* spp.) in the *ND2* gene [90], in lineages of an Australian bird (*Eopsaltria australis*) [20] in *ND4* and *ND4L*. Similarly the Chinese snub-nosed monkey (*Rinopithecus* ssp.) showed evidence of adaptive variation related with physiological hypoxia and cold stress in altitude in *ND2* and *ND6* [91]. Few studies have assessed if selection on mtDNA genes is correlated with environmental variables [92]. In the Atlantic herring (*Clupea harengus*), significant positive selection was observed in *ND2*, *ND4* and *ND5*. Correlations between genetic diversity and variation of environmental factors (temperature, salinity and latitude) were generally weak, although the authors addressed some correlation between temperature and genetic distances with *ND3* and *ND4L* [92].

#### ATP synthase

In our study, the Ka/Ks ratio for *ATP8* was lower than 1, suggesting negative selection for the gene, although it was higher than any other value for mtDNA genes in penguins. However, one codon of *ATP8* (Complex V) showed evidence of positive selection (under model FEL). Complex V (or ATP synthase) is the final enzyme in the OXPHOS pathway and is ubiquitous throughout all forms of life and biological functions. The enzyme uses the energy stored in a proton gradient across a membrane to drive the synthesis of ATP from ADP and phosphate. Complex V uses the proton gradient resulting from the activity of the respiratory chain enzymes complexes I, III and IV for ATP synthesis, generating 95% of cellular ATP. Complex V is a multi-subunit complex with two functional domains, F1 and F0, for which *ATP8* encodes a core subunit of the F0 component. Across mammalian evolution, *ATP6* is more conserved, in contrast to the *ATP8* subunit which has some highly variable sites with extreme amino acid changes of properties, suggesting that there are differences in its regulatory function among species [88].

#### Cytochrome c oxidase

*COXI,* complex IV, showed purifying selection in penguins, as has been observed across all Aves [32]. We estimated Ka/Ks ratios close to zero for all comparison among species and genera, and similar results were obtained using both the SLAC and FEL methods. These genes from complex IV represent the terminal complex of the respiratory chain. It catalyzes electron transfer from cytochrome c to molecular oxygen, reducing the latter to water. It has been postulated that Complex IV of the OXPHOS, in which *COX1* is one of the two catalytic subunits, has the property of intrinsic uncoupling [93], through which it regulates and increases the coupling efficiency to produce ATP or heat. This mechanism is consistent with the negative selection observed for this gene, as adaptations can occur by changing the configuration of the protein, a mechanism that does not occur in complexes I and V.

Further studies are required to completely understand the factors constraining the evolutionary rate of mitogenomes in penguins. Evolution of protein sequences depends on functional constraints including differential gene expression, protein folding and connectivity, functional importance, and expression breadth among tissues [94]. Here, we discussed the implications of functionality of different mtDNA genes on the evolutionary rate of a protein. Alternatively, Zang and Yang [94], suggest that this rate may be predominantly influenced by its expression level rather than functional importance.

### Mitogenomics and ecological-evolutionary hypotheses to explain selection

There are two hypotheses generally offered to explain patterns of mitochondrial genome evolution. The first is that temperature is a primary driver of selection and the evolution of mtDNA genes. For penguins, speciation and adaptation are seemingly strongly correlated with temperature differences of ocean water between the Antarctic and the tropics [35–38]. The mitochondrial OXPHOS generates both energy and heat, with the balance of each depending on the coupling efficiency of the electron transport chain to the *ATP* synthase activity [2, 3]. Less efficient energy production and more heat production would be beneficial for survival in cold environmental conditions [2]. A reduced proton leak would lead to incremental cold sensitivity that effectively constrains the biogeographic distribution of species with this pattern of mtDNA variation [4, 5]. Selection on mtDNA variation influencing mitochondrial bioenergetics would thus be plausible drivers of adaptation and speciation [6], and indeed we detected correlation between Ka/Ks from ND4 and sea suface water temperature which should be further investigated.

A second hypothesis is that the risk of starvation associated with restricted food availability (less abundant or less reliable food source) drives mtDNA gene evolution, since starvation can generate more efficient coupling of energy production by the OXPHOS pathway [7]. Three mechanisms have been suggested for the evolution of starvation resistance and each may be influenced by mitochondrial diversity: (a) greater energy reserves (lipid stores); (b) reduced rate at which reserves are utilized; (c) and lowering of the minimal resources required for survival [7, 9]. Reducing energy requirements may occur by reducing ROS and heat production and thus increasing OXPHOS efficiency [8, 10]. Chl can be considered to be a measure or correlate of primary productivity and therefore resource availability in the Antarctic marine food web. Penguins as top predators, are dependent on the productivity of lower trophic levels. Therefore, the high variation in productivity of the habitats throughout the *Spheniscus* species distributional ranges may have led to the evolution of more efficient coupling in those species, compared to the stable environments of Emperor and Adélie penguins. For example, high variability in levels of Chl is observed within the range of *S. humboldti*, and becomes further exacerbated during El Niño events, leading to a higher probability of mortality due to starvation. In this circumstance, selection may favor OXPHOS efficiency rather than heat production. During El Niño events, the upwelling of nutrients is interrupted, reducing food availability and increasing water temperature in Chilean-Peruvian waters [95]. This affects fish abundance, causing increased Galapagos and Humboldt penguin mortality, nest desertion and large-scale movements [40, 42, 96]. Although Chl could be an important variable for mtDNA evolution, only a weak correlation between Ka/Ks ratio of *COXI* and Chl across small geographic scales was detected here. Therefore, our results should be further investigated in penguins not only at a species level, but also in populations of each penguin species across a gradient of sea surface temperatures (e.g. across latitudes) and chlorophyll levels.

Although *ATP8* was not correlated to SST and Chl here, the Ka/Ks ratio values were higher than in any other gene compared. Moreover, it showed a remarkable pattern for all comparisons among species distributed in extremely different environments (tropical and hot temperate climate versus polar climate). We found contrasting results for *ATP8*. *A. forsteri* and *Spheniscus* had the lowest values for *ATP8* genes in pairwise comparisons among all genera. This may be indicative of different mechanisms of adaptation among genes and species depending on their individual habitat requirements. However, we cannot discount the possibility that this gene is associated with other selective variables, which in turn are correlated with climatic and productive variations observed in the distributional ranges of penguins. Indeed, it was recently suggested that evolution of mitochondrial genes might be linked with pathogen variability [11].

An increasing number of studies have implicated mitochondria with innate immune responses, including the control of apoptotic signaling, sterile inflammation, and antibacterial and antiviral response [12–15]. Although it is uncertain what direct role mitochondrial encoded genes have in immune response, it bears further evaluation. Antarctica and the tropics differ considerably in virulence, ensemble, abundance, development and diversity of pathogens. Just recently, the influenza virus was detected in penguins in Antarctica [97–99]. Antarctic wildlife is particularly susceptible to infectious agents because of its geographic isolation and extreme environment, which increases the likelihood that the fauna has evolved under low “pathogen pressure” and are thus immunologically naïve [11].

### Implications for future adaptation to changing environments

Ongoing human-induced climatic changes are predicted to have large impacts on the world biota, including penguin species [44–46, 100]. *Pygoscelis* species appear to be changing their distributions. *P. papua* populations have been increasing along the Antarctic Peninsula in association with an abrupt increase of water temperatures, while populations of *P. adeliae* and *P. antarcticus* have been decreasing [44–46]. The decline and disappearance of an *A. forsteri* colony was associated with the rise in local mean annual air temperature and decline in seasonal sea ice duration [101]. *S. magellanicus* populations have experienced increasing reproductive failure, measured in chicks’ mortality associated with starvation, rain, heat, and predation, which has been associated with climate change and an increase in frequency and intensity of storms [102]. *S. humboldti* and *S. mendiculus* have been historically affected by strong El Niño events causing high mortality, nesting delay, nest flooding and breeding failure [40–42, 96, 103]. El Niño events are becoming more intense and frequent due to global climate change [104, 105]. The decreasing food availability for penguins in South America during El Niño events [95], and for penguins in Antarctica during the past 50 years [106] has caused not only direct mortality. Increased mortality has also been attributed to compromised immune systems, since adequate nutrition is fundamental to maintaining a functioning immune system [107]. The expanding human activities in Antarctica also increase the risk of introduction of infectious diseases into this naïve wildlife community [108]. Therefore, selective pressures on mtDNA genes by environmental factors such as temperature, food availability and novel pathogens could intensify significantly with climate change.

The effect of climate change on gene evolution does not have to be direct. As penguins are top marine predators, changes at all trophic levels will affect penguin population demography [109]. However, understanding the genes involved in adaptation to climate by penguin species may permit us to predict how the species are physiologically limited and how they may be able to respond to environmental changes such as climate warming.

## Conclusions

Here, we identify mtDNA candidate genes under selection which could be involved in broad-scale adaptations of penguins to their environment. Our results suggest a strong negative selection for *COX1,* and a positive selection at codon sites on *ATP8* and *ND4*. We also found a high correlation between *ND4* and sea surface temperature. Further studies on these genes at the intraspecific level are required to completely understand penguin adaptation across different environmental conditions. This is a novel study that illustrates the applicability of large-scale remote sensing as a useful approach to comprehend adaptation to the environment occurring at a molecular level. Integration of these different data provide insights into how penguins sepcies have adapted to their enviornments and therefore how they may respond to future, human-induced changes to their environment.

## Additional files

Additional file 1: Table S1. Pairwise Ka/Ks comparison of sequence divergence for the 13 mitochondria protein-coding genes. (DOCX 44 kb)

Additional file 2: Table S2. Estimates of Evolutionary Divergence between Sequences. (DOCX 21 kb)

Additional file 3: Table S3. Example of matrix between the environmental distance (ENDIST), chlorophyll (CHL) in this example, and the the Ka/Ks. Then environmental data (CHL) were grouped in three class distances for the GLM and Mantel tests. (DOCX 35 kb)

Additional file 4: Table S4. Significant values of GLM and Mantel Test for Ka/Ks ratio and nucleotide diversity (*π*) for each gene and environmental data (SST, CHL, COM) at different class distance. (DOCX 26 kb)
