# Supplementary material for: Landscape genomics: natural selection drives the evolution of mitogenome in penguins
Source: BMC Genomics. 2018 Jan 16;19:53. doi: 10.1186/s12864-017-4424-9 (PMC5771141; doi:10.1186/s12864-017-4424-9)
Supplement: Supplementary file 1 — Pairwise Ka/Ks comparison of sequence divergence for the 13 mitochondria protein-coding genes. (DOCX 44 kb) [file 12864_2017_4424_MOESM1_ESM.docx]

**Additional file S1.** **Pairwise Ka/Ks comparison of sequence divergence for the 13 mitochondria protein-coding genes.**

|  | **ND1** | **ND2** | **COX1** | **COX2** | **ATP8** | **ATP6** | **COX3** | **ND3** | **ND4L** | **ND4** | **ND5** | **CYTB** | **ND6** |
| --- | --- | --- | --- | --- | --- | --- | --- | --- | --- | --- | --- | --- | --- |
| Sh x Sme | 0.0345 | 0.1199 | N | N | N | N | 0.0000 | N | N | 0.2439 | 0.0648 | 0.1778 | N |
| Sh x Sd | 0.0596 | 0.1040 | 0.0000 | 0.0000 | 0.0000 | 0.1462 | 0.0417 | 0.0000 | 0.0000 | 0.0373 | 0.1014 | 0.1317 | 0.0579 |
| Sh x Sma | 0.0657 | 0.1343 | 0.0000 | 0.0387 | 0.1470 | 0.1990 | 0.0864 | 0.0000 | 0.0000 | 0.0691 | 0.1039 | 0.1059 | 0.3750 |
| Sme X Sd | 0.0503 | 0.0523 | 0.0000 | 0.0000 | 0.0000 | 0.1462 | 0.0485 | 0.0000 | 0.0000 | 0.0598 | 0.1001 | 0.1059 | 0.0579 |
| Sme x Sma | 0.0546 | 0.0979 | 0.0000 | 0.0387 | 0.1470 | 0.1990 | 0.1065 | 0.0000 | 0.0000 | 0.0890 | 0.0965 | 0.0742 | 0.3750 |
| Sd x Sma | 0.0000 | 0.1145 | 0.0000 | 0.0625 | 0.3000 | 0.0510 | 0.0320 | 0.0000 | 0.0000 | 0.0797 | 0.1423 | 0.1366 | 0.0724 |
| **Sphen** | **0.0441** | **0.1038** | **0.0000** | **0.0280** | **0.1188** | **0.1483** | **0.0525** | **0.0000** | **0.0000** | **0.0965** | **0.1015** | **0.1220** | **0.1876** |
| Pan x Ppa | 0.0343 | 0.1225 | 0.0092 | 0.0550 | 0.2480 | 0.0285 | 0.0264 | 0.0991 | 0.1056 | 0.0609 | 0.1700 | 0.0578 | 0.1045 |
| Pan x Pad | 0.0298 | 0.1294 | 0.0104 | 0.0145 | 0.0653 | 0.0436 | 0.0330 | 0.0769 | 0.0203 | 0.0519 | 0.1067 | 0.0340 | 0.0538 |
| Ppa x Pad | 0.0480 | 0.1057 | 0.0070 | 0.0408 | 0.1238 | 0.0411 | 0.0248 | 0.0604 | 0.0462 | 0.0643 | 0.1192 | 0.0332 | 0.0581 |
| **Pygos** | **0.0373** | **0.1192** | **0.0088** | **0.0367** | **0.1457** | **0.0377** | **0.0280** | **0.0788** | **0.0574** | **0.0590** | **0.1320** | **0.0416** | **0.0721** |
| Sme x Pan | 0.0407 | 0.0718 | 0.0100 | 0.0180 | 0.0922 | 0.0592 | 0.0366 | 0.0919 | 0.0841 | 0.0906 | 0.1055 | 0.0634 | 0.0684 |
| Sme x Ppa | 0.0488 | 0.0919 | 0.0079 | 0.0265 | 0.1450 | 0.0563 | 0.0352 | 0.0613 | 0.0944 | 0.0804 | 0.0943 | 0.0850 | 0.0743 |
| Sme x Pad | 0.0483 | 0.1159 | 0.0114 | 0.0158 | 0.1371 | 0.0588 | 0.0414 | 0.0615 | 0.1001 | 0.0795 | 0.0853 | 0.0767 | 0.0773 |
| Sh x Pan | 0.0417 | 0.0673 | 0.0100 | 0.0180 | 0.0922 | 0.0592 | 0.0350 | 0.0919 | 0.0841 | 0.0845 | 0.1037 | 0.0682 | 0.0684 |
| Sh x Ppa | 0.0451 | 0.0829 | 0.0079 | 0.0265 | 0.1450 | 0.0563 | 0.0336 | 0.0613 | 0.0944 | 0.0742 | 0.0960 | 0.0923 | 0.0743 |
| Sh x Pad | 0.0561 | 0.1046 | 0.0114 | 0.0158 | 0.1371 | 0.0588 | 0.0394 | 0.0615 | 0.1001 | 0.0743 | 0.0850 | 0.0821 | 0.0773 |
| Sma x Pan | 0.0334 | 0.0727 | 0.0107 | 0.0289 | 0.0949 | 0.0665 | 0.0276 | 0.0963 | 0.0798 | 0.0824 | 0.1244 | 0.0670 | 0.0714 |
| Sma x Ppa | 0.0370 | 0.0950 | 0.0084 | 0.0372 | 0.1435 | 0.0603 | 0.0250 | 0.0641 | 0.0895 | 0.0665 | 0.1107 | 0.0869 | 0.0774 |
| Sma x Pad | 0.0436 | 0.1203 | 0.0121 | 0.0249 | 0.1385 | 0.0534 | 0.0312 | 0.0642 | 0.0949 | 0.0766 | 0.1026 | 0.0793 | 0.0841 |
| Sd x Pan | 0.0298 | 0.0664 | 0.0104 | 0.0215 | 0.0922 | 0.0652 | 0.0330 | 0.0768 | 0.0846 | 0.0833 | 0.1204 | 0.0679 | 0.0745 |
| Sd x Ppa | 0.0339 | 0.0879 | 0.0083 | 0.0292 | 0.1450 | 0.0568 | 0.0311 | 0.0540 | 0.0952 | 0.0758 | 0.1110 | 0.0923 | 0.0829 |
| Sd x Sad | 0.0436 | 0.1126 | 0.0120 | 0.0175 | 0.1371 | 0.0647 | 0.0392 | 0.0541 | 0.1006 | 0.0809 | 0.1046 | 0.0864 | 0.0842 |
| **Sphen x Pygos** | **0.0418** | **0.0908** | **0.0100** | **0.0233** | **0.1250** | **0.0596** | **0.0340** | **0.0699** | **0.0918** | **0.0791** | **0.1036** | **0.0790** | **0.0762** |
| **Em x Eu** | **0.0409** | **0.1125** | **0.0101** | **0.0102** | **0.3099** | **0.0929** | **0.0150** | **0.0527** | **0.0819** | **0.0613** | **0.1099** | **0.0562** | **0.0938** |
| Em x Sma | 0.0570 | 0.0796 | 0.0173 | 0.0215 | 0.3845 | 0.0598 | 0.0298 | 0.0565 | 0.0565 | 0.0522 | 0.1083 | 0.0461 | 0.0626 |
| Em x Sde | 0.0570 | 0.0687 | 0.0175 | 0.0124 | 0.4116 | 0.0724 | 0.0227 | 0.0729 | 0.0604 | 0.0486 | 0.1003 | 0.0544 | 0.0695 |
| Em x Shu | 0.0597 | 0.0634 | 0.0156 | 0.0128 | 0.4116 | 0.0811 | 0.0237 | 0.0815 | 0.0601 | 0.0523 | 0.0992 | 0.0504 | 0.0572 |
| Em x Sme | 0.0531 | 0.0830 | 0.0156 | 0.0128 | 0.4116 | 0.0811 | 0.0250 | 0.0865 | 0.0601 | 0.0568 | 0.0984 | 0.0434 | 0.0572 |
| **Em x Sphen** | **0.0567** | **0.0737** | **0.0165** | **0.0149** | **0.4048** | **0.0736** | **0.0253** | **0.0743** | **0.0593** | **0.0525** | **0.1016** | **0.0486** | **0.0616** |
| Em x Pan | 0.0329 | 0.0774 | 0.0151 | 0.0269 | 0.2421 | 0.0817 | 0.0365 | 0.0952 | 0.0766 | 0.1054 | 0.1069 | 0.0559 | 0.0801 |
| Em x Ppa | 0.0476 | 0.0891 | 0.0104 | 0.0333 | 0.2423 | 0.0670 | 0.0320 | 0.0489 | 0.0641 | 0.0789 | 0.1071 | 0.0665 | 0.0904 |
| Em x Pad | 0.0341 | 0.0850 | 0.0141 | 0.0239 | 0.2897 | 0.0696 | 0.0394 | 0.0636 | 0.0742 | 0.0815 | 0.1219 | 0.0615 | 0.0826 |
| **Em x Pygos** | **0.0382** | **0.0838** | **0.0132** | **0.0280** | **0.2581** | **0.0727** | **0.0360** | **0.0692** | **0.0716** | **0.0886** | **0.1119** | **0.0613** | **0.0844** |
| Ech x Sma | 0.0458 | 0.1235 | 0.0097 | 0.0060 | 0.2592 | 0.0650 | 0.0239 | 0.1017 | 0.0884 | 0.0432 | 0.0901 | 0.0592 | 0.0629 |
| Ech x Sde | 0.0435 | 0.1185 | 0.0093 | 0.0000 | 0.2682 | 0.0545 | 0.0171 | 0.0921 | 0.0892 | 0.0450 | 0.0857 | 0.0566 | 0.0627 |
| Ech x Shu | 0.0481 | 0.1223 | 0.0084 | 0.0000 | 0.2137 | 0.0445 | 0.0179 | 0.0968 | 0.0999 | 0.0502 | 0.0806 | 0.0640 | 0.0556 |
| Ech x Sme | 0.0519 | 0.1272 | 0.0084 | 0.0000 | 0.2137 | 0.0445 | 0.0179 | 0.0968 | 0.0999 | 0.0568 | 0.0778 | 0.0552 | 0.0556 |
| **Ech x Sphen** | **0.0473** | **0.1229** | **0.0089** | **0.0015** | **0.2387** | **0.0521** | **0.0192** | **0.0969** | **0.0944** | **0.0488** | **0.0836** | **0.0587** | **0.0592** |
| Ech x Pan | 0.0176 | 0.0839 | 0.0115 | 0.0141 | 0.1392 | 0.0868 | 0.0276 | 0.0977 | 0.0683 | 0.0833 | 0.1249 | 0.0738 | 0.1263 |
| Ech x Ppa | 0.0316 | 0.1054 | 0.0054 | 0.0263 | 0.1636 | 0.0719 | 0.0178 | 0.0644 | 0.0810 | 0.0786 | 0.1063 | 0.0858 | 0.1742 |
| Ech x Pad | 0.0334 | 0.1060 | 0.0104 | 0.0125 | 0.2649 | 0.0761 | 0.0272 | 0.0570 | 0.0829 | 0.0710 | 0.1038 | 0.0745 | 0.1465 |
| **Ech x Pygos** | **0.0275** | **0.0984** | **0.0091** | **0.0176** | **0.1892** | **0.0783** | **0.0242** | **0.0731** | **0.0774** | **0.0776** | **0.1117** | **0.0780** | **0.1490** |
| Apt x Shu | 0.0491 | 0.1083 | 0.0123 | 0.0148 | 0.1565 | 0.0730 | 0.0238 | 0.1174 | 0.0525 | 0.0901 | 0.1146 | 0.0495 | 0.0746 |
| Apt x Sma | 0.0390 | 0.1051 | 0.0131 | 0.0234 | 0.1559 | 0.0938 | 0.0096 | 0.1114 | 0.0553 | 0.0818 | 0.1309 | 0.0493 | 0.0812 |
| Apt x Sme | 0.0469 | 0.1093 | 0.0123 | 0.0148 | 0.1565 | 0.0730 | 0.0244 | 0.1174 | 0.0525 | 0.0961 | 0.1175 | 0.0444 | 0.0746 |
| Apt x Sde | 0.0402 | 0.1037 | 0.0129 | 0.0152 | 0.1417 | 0.0895 | 0.0151 | 0.1008 | 0.0528 | 0.0901 | 0.1314 | 0.0553 | 0.0666 |
| **Apt x Sphen** | **0.0438** | **0.1066** | **0.0127** | **0.0171** | **0.1526** | **0.0823** | **0.0182** | **0.1117** | **0.0533** | **0.0895** | **0.1236** | **0.0496** | **0.0743** |
| Apt x Pan | 0.0221 | 0.0795 | 0.0057 | 0.0331 | 0.1412 | 0.0878 | 0.0242 | 0.1056 | 0.0764 | 0.0908 | 0.1527 | 0.0470 | 0.0886 |
| Apt x Ppa | 0.0308 | 0.0977 | 0.0073 | 0.0189 | 0.1778 | 0.0905 | 0.0223 | 0.0916 | 0.0863 | 0.0622 | 0.1457 | 0.0559 | 0.0911 |
| Apt x Pad | 0.0197 | 0.1049 | 0.0083 | 0.0264 | 0.2218 | 0.0935 | 0.0277 | 0.0447 | 0.0750 | 0.0747 | 0.1315 | 0.0391 | 0.0739 |
| **Apt x Pygos** | **0.0242** | **0.0940** | **0.0071** | **0.0261** | **0.1803** | **0.0906** | **0.0247** | **0.0806** | **0.0792** | **0.0759** | **0.1433** | **0.0473** | **0.0845** |
| **Apt x Em** | **0.0481** | **0.0902** | **0.0193** | **0.0203** | **0.3735** | **0.0897** | **0.0287** | **0.0872** | **0.0641** | **0.0817** | **0.1391** | **0.0528** | **0.0787** |
| **Apt x Ech** | **0.0320** | **0.1100** | **0.0149** | **0.0114** | **0.2613** | **0.0782** | **0.0248** | **0.0834** | **0.0855** | **0.0831** | **0.1361** | **0.0731** | **0.0911** |

*Spheniscus* (Sphen): *S. humboldt* (Shu), *S. magellanicus* (Sma), *S. mendiculus* (Sme), *S. demersus* (Sde), *Pygoscelis* ssp.(Pygos): *P. antarcticus* (Pan), *P. papua* (Ppa), *P. adelie* (Pad), *Eudyptula minor* (Emi), *Eudyptes chrysocome* (Ech) and *Aptenodytes forsteri* (Apt). N is indicated when KS = 0.
