# Supplementary material for: Landscape genomics: natural selection drives the evolution of mitogenome in penguins
Source: BMC Genomics. 2018 Jan 16;19:53. doi: 10.1186/s12864-017-4424-9 (PMC5771141; doi:10.1186/s12864-017-4424-9)
Supplement: Supplementary file 2 — Estimates of Evolutionary Divergence between Sequences. (DOCX 21 kb) [file 12864_2017_4424_MOESM2_ESM.docx]

**S2. Estimates of Evolutionary Divergence between Sequences.**

|  | 1 | 2 | 3 | *4* | 5 | 6 | *7* | *8* | *9* | *10* | 11 | 12 | 13 | 14 | 15 |
| --- | --- | --- | --- | --- | --- | --- | --- | --- | --- | --- | --- | --- | --- | --- | --- |
| *1. S. magellanicus* (PD) |  |  |  |  |  |  |  |  |  |  |  |  |  |  |  |
| *2. S. magellanicus* (MI) | 0.001 |  |  |  |  |  |  |  |  |  |  |  |  |  |  |
| *3. S. magellanicus* (CI) | 0.001 | 0.001 |  |  |  |  |  |  |  |  |  |  |  |  |  |
| *4. S. demersus* | 0.010 | 0.010 | 0.010 |  |  |  |  |  |  |  |  |  |  |  |  |
| *5. S. humboldti* (CA) | 0.015 | 0.015 | 0.015 | 0.015 |  |  |  |  |  |  |  |  |  |  |  |
| *6. S. humboldti* (SJ) | 0.015 | 0.015 | 0.015 | 0.015 | 0.002 |  |  |  |  |  |  |  |  |  |  |
| *7. S. mendiculus* | 0.015 | 0.015 | 0.015 | 0.015 | 0.004 | 0.005 |  |  |  |  |  |  |  |  |  |
| *8. E. minor* | 0.071 | 0.071 | 0.071 | 0.071 | 0.071 | 0.071 | 0.071 |  |  |  |  |  |  |  |  |
| *9. E. chrysocome* | 0.073 | 0.073 | 0.072 | 0.073 | 0.072 | 0.073 | 0.072 | 0.076 |  |  |  |  |  |  |  |
| *10. A. forsteri* | 0.089 | 0.089 | 0.089 | 0.090 | 0.091 | 0.091 | 0.090 | 0.094 | 0.092 |  |  |  |  |  |  |
| *11. P. antarctica* (NB) | 0.090 | 0.090 | 0.090 | 0.091 | 0.091 | 0.092 | 0.091 | 0.096 | 0.090 | 0.091 |  |  |  |  |  |
| *12. P. antarctica* (KO) | 0.090 | 0.090 | 0.090 | 0.091 | 0.091 | 0.092 | 0.091 | 0.095 | 0.089 | 0.091 | 0.000 |  |  |  |  |
| *13. P. papua* (GGV) | 0.090 | 0.091 | 0.090 | 0.091 | 0.090 | 0.091 | 0.090 | 0.095 | 0.088 | 0.090 | 0.047 | 0.047 |  |  |  |
| *14. P. adelie* (AB) | 0.090 | 0.090 | 0.090 | 0.090 | 0.091 | 0.092 | 0.091 | 0.097 | 0.092 | 0.092 | 0.059 | 0.059 | 0.061 |  |  |
| *15. P. adelie* (RS) | 0.090 | 0.091 | 0.090 | 0.090 | 0.091 | 0.092 | 0.091 | 0.097 | 0.092 | 0.093 | 0.059 | 0.059 | 0.061 | 0.000 |  |

The number of base substitutions per site from between sequences are shown.PD: Puerto Deseado. MI: Magdalena Island. CI: Chiloé Island. CA: Cachagua. SJ: Punta San Juan. NB: Narebski Base. KO: Kopaitic. GGV: Gabriel González Videla Base. AB: Arctowski Base. RS: Ross Sea
