# Supplementary material for: Landscape genomics: natural selection drives the evolution of mitogenome in penguins
Source: BMC Genomics. 2018 Jan 16;19:53. doi: 10.1186/s12864-017-4424-9 (PMC5771141; doi:10.1186/s12864-017-4424-9)
Supplement: Supplementary file 3 — Example of matrix between the environmental distance (ENDIST), chlorophyll (CHL) in this example, and the the Ka/Ks. Then environmental data (CHL) were grouped in three class distances for the GLM and Mantel tests. (DOCX 35 kb) [file 12864_2017_4424_MOESM3_ESM.docx]

S3. Example of matrix between the environmental distance (ENDIST), chlorophyll (CHL) in this example, and the the Ka/Ks.

Then environmental data (CHL) were grouped in three class distances for the GLM and Mantel tests.

| Pairwise species | ENDIST (CHL) | Class Distance | ND1 | ND2 | COX1 | COX2 | ATP8 | ATP6 | COX3 | ND3 | ND4L | ND4 | ND5 | CYTB | ND6 |
| --- | --- | --- | --- | --- | --- | --- | --- | --- | --- | --- | --- | --- | --- | --- | --- |
| Apt x Pad | 0.089317121 | 0-33 | 0.02 | 0.105 | 0.008 | 0.026 | 0.222 | 0.093 | 0.028 | 0.045 | 0.075 | 0.075 | 0.131 | 0.039 | 0.074 |
| Apt x Ppa | 0.15745609 |  | 0.031 | 0.098 | 0.007 | 0.019 | 0.178 | 0.09 | 0.022 | 0.092 | 0.086 | 0.062 | 0.146 | 0.056 | 0.091 |
| Pa x Ppa | 0.176444475 |  | 0.034 | 0.122 | 0.009 | 0.055 | 0.248 | 0.029 | 0.026 | 0.099 | 0.106 | 0.061 | 0.17 | 0.058 | 0.105 |
| Em x Pa | 0.178444912 |  | 0.033 | 0.077 | 0.015 | 0.027 | 0.242 | 0.082 | 0.037 | 0.095 | 0.077 | 0.105 | 0.107 | 0.056 | 0.08 |
| Ech x Sme | 0.202993536 |  | 0.052 | 0.127 | 0.008 | 0 | 0.214 | 0.045 | 0.018 | 0.097 | 0.1 | 0.057 | 0.078 | 0.055 | 0.056 |
| Em x Ppa | 0.205092947 |  | 0.048 | 0.089 | 0.01 | 0.033 | 0.242 | 0.067 | 0.032 | 0.049 | 0.064 | 0.079 | 0.107 | 0.067 | 0.09 |
| Apt x Pa | 0.20815722 |  | 0.022 | 0.079 | 0.006 | 0.033 | 0.141 | 0.088 | 0.024 | 0.106 | 0.076 | 0.091 | 0.153 | 0.047 | 0.089 |
| Ppa x Pad | 0.227843365 |  | 0.048 | 0.106 | 0.007 | 0.041 | 0.124 | 0.041 | 0.025 | 0.06 | 0.046 | 0.064 | 0.119 | 0.033 | 0.058 |
| Ech x Ppa | 0.250853185 |  | 0.032 | 0.105 | 0.005 | 0.026 | 0.164 | 0.072 | 0.018 | 0.064 | 0.081 | 0.079 | 0.106 | 0.086 | 0.174 |
| Sme x Ppa | 0.275110983 |  | 0.049 | 0.092 | 0.008 | 0.027 | 0.145 | 0.056 | 0.035 | 0.061 | 0.094 | 0.08 | 0.094 | 0.085 | 0.074 |
| Pa x Pad | 0.275195315 |  | 0.03 | 0.129 | 0.01 | 0.014 | 0.065 | 0.044 | 0.033 | 0.077 | 0.02 | 0.052 | 0.107 | 0.034 | 0.054 |
| Apt x Em | 0.304854432 |  | 0.048 | 0.09 | 0.019 | 0.02 | 0.374 | 0.09 | 0.029 | 0.087 | 0.064 | 0.082 | 0.139 | 0.053 | 0.079 |
| Em x Sme | 0.324025894 |  | 0.053 | 0.083 | 0.016 | 0.013 | 0.412 | 0.081 | 0.025 | 0.086 | 0.06 | 0.057 | 0.098 | 0.043 | 0.057 |
| Apt x Ech | 0.350894865 |  | 0.032 | 0.11 | 0.015 | 0.011 | 0.261 | 0.078 | 0.025 | 0.083 | 0.086 | 0.083 | 0.136 | 0.073 | 0.091 |
| Em x Pad | 0.375952351 |  | 0.034 | 0.085 | 0.014 | 0.024 | 0.29 | 0.07 | 0.039 | 0.064 | 0.074 | 0.082 | 0.122 | 0.061 | 0.083 |
| Ech x Pad | 0.378421883 | 33-66 | 0.033 | 0.106 | 0.01 | 0.013 | 0.265 | 0.076 | 0.027 | 0.057 | 0.083 | 0.071 | 0.104 | 0.075 | 0.147 |
| Sme x Pa | 0.380948396 |  | 0.041 | 0.072 | 0.01 | 0.018 | 0.092 | 0.059 | 0.037 | 0.092 | 0.084 | 0.091 | 0.106 | 0.063 | 0.068 |
| Em x Eu | 0.384232392 |  | 0.041 | 0.112 | 0.01 | 0.01 | 0.31 | 0.093 | 0.015 | 0.053 | 0.082 | 0.061 | 0.11 | 0.056 | 0.094 |
| Apt x Sme | 0.399001103 |  | 0.047 | 0.109 | 0.012 | 0.015 | 0.156 | 0.073 | 0.024 | 0.117 | 0.052 | 0.096 | 0.118 | 0.044 | 0.075 |
| Ech x Pa | 0.419849072 |  | 0.018 | 0.084 | 0.011 | 0.014 | 0.139 | 0.087 | 0.028 | 0.098 | 0.068 | 0.083 | 0.125 | 0.074 | 0.126 |
| Sme x Pad | 0.42810594 |  | 0.048 | 0.116 | 0.011 | 0.016 | 0.137 | 0.059 | 0.041 | 0.061 | 0.1 | 0.08 | 0.085 | 0.077 | 0.077 |
| Sd x Sma | 0.487647619 |  | 0 | 0.115 | 0 | 0.063 | 0.3 | 0.051 | 0.032 | 0 | 0 | 0.08 | 0.142 | 0.137 | 0.072 |
| Ech x Sma | 0.644624763 |  | 0.046 | 0.124 | 0.01 | 0.006 | 0.259 | 0.065 | 0.024 | 0.102 | 0.088 | 0.043 | 0.09 | 0.059 | 0.063 |
| Shu x Sd | 0.706973842 |  | 0.06 | 0.104 | 0 | 0 | 0 | 0.146 | 0.042 | 0 | 0 | 0.037 | 0.101 | 0.132 | 0.058 |
| Sme x Sma | 0.72854282 |  | 0.055 | 0.098 | 0 | 0.039 | 0.147 | 0.199 | 0.106 | 0 | 0 | 0.089 | 0.096 | 0.074 | 0.375 |
| Shu x Sma | 0.866824277 |  | 0.066 | 0.134 | 0 | 0.039 | 0.147 | 0.199 | 0.086 | 0 | 0 | 0.069 | 0.104 | 0.106 | 0.375 |
| Sma x Ppa | 0.887774053 |  | 0.037 | 0.095 | 0.008 | 0.037 | 0.144 | 0.06 | 0.025 | 0.064 | 0.089 | 0.067 | 0.111 | 0.087 | 0.077 |
| Sma x Pad | 0.945541553 |  | 0.044 | 0.12 | 0.012 | 0.025 | 0.138 | 0.053 | 0.031 | 0.064 | 0.095 | 0.077 | 0.103 | 0.079 | 0.084 |
| Apt x Sma | 0.954941361 |  | 0.039 | 0.105 | 0.013 | 0.023 | 0.156 | 0.094 | 0.01 | 0.111 | 0.055 | 0.082 | 0.131 | 0.049 | 0.081 |
| Sme X Sd | 1.013337684 | 66-100 | 0.05 | 0.052 | 0 | 0 | 0 | 0.146 | 0.049 | 0 | 0 | 0.06 | 0.1 | 0.106 | 0.058 |
| Ech x Sde | 1.01491049 |  | 0.044 | 0.118 | 0.009 | 0 | 0.268 | 0.055 | 0.017 | 0.092 | 0.089 | 0.045 | 0.086 | 0.057 | 0.063 |
| Em x Sma | 1.016819602 |  | 0.057 | 0.08 | 0.017 | 0.021 | 0.384 | 0.06 | 0.03 | 0.056 | 0.057 | 0.052 | 0.108 | 0.046 | 0.063 |
| Sma x Pa | 1.050119857 |  | 0.033 | 0.073 | 0.011 | 0.029 | 0.095 | 0.066 | 0.028 | 0.096 | 0.08 | 0.082 | 0.124 | 0.067 | 0.071 |
| Sd x Sad | 1.241166889 |  | 0.044 | 0.113 | 0.012 | 0.018 | 0.137 | 0.065 | 0.039 | 0.054 | 0.101 | 0.081 | 0.105 | 0.086 | 0.084 |
| Sd x Pa | 1.311068276 |  | 0.03 | 0.066 | 0.01 | 0.021 | 0.092 | 0.065 | 0.033 | 0.077 | 0.085 | 0.083 | 0.12 | 0.068 | 0.075 |
| Apt x Sde | 1.32364433 |  | 0.04 | 0.104 | 0.013 | 0.015 | 0.142 | 0.089 | 0.015 | 0.101 | 0.053 | 0.09 | 0.131 | 0.055 | 0.067 |
| Em x Sde | 1.333467919 |  | 0.057 | 0.069 | 0.017 | 0.012 | 0.412 | 0.072 | 0.023 | 0.073 | 0.06 | 0.049 | 0.1 | 0.054 | 0.069 |
| Sd x Ppa | 1.376100554 |  | 0.034 | 0.088 | 0.008 | 0.029 | 0.145 | 0.057 | 0.031 | 0.054 | 0.095 | 0.076 | 0.111 | 0.092 | 0.083 |
| Ech x Shu | 1.497236988 |  | 0.048 | 0.122 | 0.008 | 0 | 0.214 | 0.045 | 0.018 | 0.097 | 0.1 | 0.05 | 0.081 | 0.064 | 0.056 |
| Shu x Ppa | 1.726406176 |  | 0.045 | 0.083 | 0.008 | 0.027 | 0.145 | 0.056 | 0.034 | 0.061 | 0.094 | 0.074 | 0.096 | 0.092 | 0.074 |
| Shu x Pad | 1.748891465 |  | 0.056 | 0.105 | 0.011 | 0.016 | 0.137 | 0.059 | 0.039 | 0.061 | 0.1 | 0.074 | 0.085 | 0.082 | 0.077 |
| Apt x Shu | 1.774267044 |  | 0.049 | 0.108 | 0.012 | 0.015 | 0.156 | 0.073 | 0.024 | 0.117 | 0.052 | 0.09 | 0.115 | 0.049 | 0.075 |
| Em x Shu | 1.868995066 |  | 0.06 | 0.063 | 0.016 | 0.013 | 0.412 | 0.081 | 0.024 | 0.082 | 0.06 | 0.052 | 0.099 | 0.05 | 0.057 |
| Shu x Pa | 1.879538716 |  | 0.042 | 0.067 | 0.01 | 0.018 | 0.092 | 0.059 | 0.035 | 0.092 | 0.084 | 0.085 | 0.104 | 0.068 | 0.068 |

*S. humboldt* (Shu), *S. magellanicus* (Sma), *S. mendiculus* (Sme), *S. demersus* (Sde), *P. antarcticus* (Pan), *P. papua* (Ppa), *P. adelie* (Pad), *Eudyptula minor* (Emi), *Eudyptes chrysocome* (Ech) and *Aptenodytes forsteri* (Apt).
