# Supplementary material for: Landscape genomics: natural selection drives the evolution of mitogenome in penguins
Source: BMC Genomics. 2018 Jan 16;19:53. doi: 10.1186/s12864-017-4424-9 (PMC5771141; doi:10.1186/s12864-017-4424-9)
Supplement: Supplementary file 4 — Significant values of GLM and Mantel Test for Ka/Ks ratio and nucleotide diversity (π) for each gene and environmental data (SST, CHL, COM) at different class distance. (DOCX 26 kb) [file 12864_2017_4424_MOESM4_ESM.docx]

S4. Significant values of GLM and Mantel Test for Ka/Ks ratio and nucleotide diversity (π) for each gene and environmental data (SST, CHL, COM) at different class distance.

| Genes | Environ. Variable | Class Distance | Ka/Ks | | | | | | π | | | | | |
| --- | --- | --- | --- | --- | --- | --- | --- | --- | --- | --- | --- | --- | --- | --- |
|  |  |  | Mantel | | | | GLM | | Mantel | | | | GLM | |
|  |  |  | *Expectation* | *Observation* | *P* | *Variance* | *AIC* | *P* | *Expectation* | *Observation* | *P* | *Variance* | *AIC* | *P* |
| ND1 | SST | 0-100 |  |  |  |  |  |  | 0.0008 | -0.0087 | 0.5261 | 0.0037 | -177.35 | 0.0361 * |
|  | CHL | 0-100 | -0.00003 | 0.0086 | 0.3758 | 0.0045 | -260.07 | 0.0185* |  |  |  |  |  |  |
|  | COM | 0-100 | -0.0007 | 0.0608 | 0.1717 | 0.0039 | -259.59 | 0.0239* |  |  |  |  |  |  |
| COX1 | SST | 0-100 |  |  |  |  |  |  | 0.0007 | 0.0121 | 0.3961 | 0.0044 | -192.14 | 0.0197 * |
|  | CHL | 0-33 | 0.0000 | 0.2216 | 0.0631 | 0.0162 | -122.01 | 0.0298* |  |  |  |  |  |  |
| ATP8 | SST | 0-100 |  |  |  |  |  |  | 0.0006 | 0.0211 | 0.3373 | 0.0039 | -145.42 | 0.0113 * |
|  | COM^1^ | 66-100 | 0.0013 | 0.2260 | 0.0953 | 0.0299 | -31.36 | 0.0487* |  |  |  |  |  |  |
|  | COM | 0-100 |  |  |  |  |  |  | 0.0003 | -0.0331 | 0.6729 | 0.0040 | -143.72 | 0.0282 * |
| ATP6 | SST | 0-100 |  |  |  |  |  |  | 0.0009 | 0.0400 | 0.2468 | 0.0038 | -178.81 | 0.0066* |
|  | COM | 66-100 |  |  |  |  |  |  | -0.0005 | 0.2196 | 0.0906 | 0.0196 | -85.30 | 0.024 * |
|  | COM | 0-100 |  |  |  |  |  |  | 0.0002 | -0.0375 | 0.6998 | 0.0042 | -175.85 | 0.0323 * |
| ND3 | COM | 33-66 | -0.0014 | 0.2642 | 0.0304* | 0.0125 | -53.22 | 0.0188* |  |  |  |  |  |  |
| ND4 | SST | 0-100 | -0.0006 | 0.2978 | 0.0001* | 0.0029 | -248.99 | 0.00001* | 0.0009 | 0.0081 | 0.4252 | 0.0041 | -182.83 | 0.0182 * |
|  | COM | 0-100 | -0.0002 | 0.0359 | 0.2392 | 0.0029 | -240.96 | 0.0195* |  |  |  |  |  |  |
| ND5 | SST | 0-100 |  |  |  |  |  |  | 0.0012 | 0.0157 | 0.3776 | 0.0042 | -175.21 | 0.0164 * |
|  | CHL^1^ | 0-100 | -0.0001 | 0.0173 | 0.3447 | 0.0044 | -220.55 | 0.0249* |  |  |  |  |  |  |
|  | COM^1^ | 0-100 | 0.0000 | 0.1312 | 0.0232* | 0.0038 | -222.34 | 0.0095* | -0.0004 | -0.0324 | 0.6552 | 0.0045 | -173.69 | 0.0377 * |
| CYTB | COM | 0-33 | -0.0008 | 0.2476 | 0.0463* | 0.0149 | -63.94 | 0.0103* |  |  |  |  |  |  |
|  | COM^1^ | 33-66 | 0.0013 | 0.4184 | 0.0064* | 0.0137 | -72.91 | 0.0112* |  |  |  |  |  |  |

*significant values; ^1^negative correlation
